# Is Increasing Diet Diversity of Animal-Source Foods Related to Better Health-Related Quality of Life among Chinese Men and Women?

**DOI:** 10.3390/nu15194183

**Published:** 2023-09-27

**Authors:** Hui Jing, Yuxin Teng, Samuel Chacha, Ziping Wang, Guoshuai Shi, Baibing Mi, Binyan Zhang, Jiaxin Cai, Yezhou Liu, Qiang Li, Yuan Shen, Jiaomei Yang, Yijun Kang, Shanshan Li, Danmeng Liu, Duolao Wang, Hong Yan, Shaonong Dang

**Affiliations:** 1 Department of Epidemiology and Biostatistics, School of Public Health, Xi’an Jiaotong University Health Science Center, Xi’an 710061, China; jingh3@stu.xjtu.edu.cn (H.J.); yx9966@stu.xjtu.edu.cn (Y.T.); chachass@stu.xjtu.edu.cn (S.C.); wzp3121115079@stu.xjtu.edu.cn (Z.W.); shiguoshuai@stu.xjtu.edu.cn (G.S.); xjtu.mi@xjtu.edu.cn (B.M.); zhangbinyan@stu.xjtu.edu.cn (B.Z.); mathcjx@stu.xjtu.edu.cn (J.C.); yezhouliu@stu.xjtu.edu.cn (Y.L.); tjlq@mail.xjtu.edu.cn (Q.L.); yulander.s@xjtu.edu.cn (Y.S.); violetyjm18@xjtu.edu.cn (J.Y.); tjkyj@xjtu.edu.cn (Y.K.); shanshancute@163.com (S.L.); danmengliu1214@163.com (D.L.); 2 Department of Clinical Sciences, Liverpool School of Tropical Medicine, Liverpool L7 8XZ, UK; wang@liverpool.ac.uk

**Keywords:** health-related quality of life, 12-Item Short Form Survey, animal-source foods, diet diversity, Chinese

## Abstract

Diet plays a crucial role in regulating individuals’ lifestyles and is closely related to health. The intake of animal-sourced foods (ASF) provides the human body with high-quality protein and various micronutrients. This study aimed to investigate whether the diversity of animal foods has a positive impact on the health-related quality of life (HRQoL) among residents. The data came from the Shaanxi baseline survey of the Northwest Chinese Regional Ethnic Cohort Study, which recruited more than 100 thousand participants aged 35 to 74 from five provinces between June 2018 and May 2019. A total of 39,997 participants in Shaanxi (mean age: 50 years; 64% women) were finally included in this current study. The animal source food diet diversity score (ASFDDS) was established based on the frequency of consuming pork, mutton, beef, poultry, seafood, eggs, pure milk, and yogurt. The physical component score (PCS) and mental component score (MCS), ranging from 0 to 100 on the 12-Item Short Form Survey (SF-12), were used to assess participants’ HRQoL. Better PCS/MCS was defined as scores higher than the 90th percentile. The results showed that men had a higher intake of ASF and ASFDDS than women. After adjusting for potential confounders, compared with those who never or rarely consumed animal foods, the likelihood of having better PCS and MCS increased by 16% (OR = 1.16, 95%CI: 1.01–1.34) and 24% (OR = 1.24, 95%CI: 1.03–1.448), respectively, in men with an ASFDDS ≥ 2. In women, a 34% increase (OR = l.34, 95%CI: 116–l.54) likelihood for better PCS was observed for an ASFDDS ≥ 2, but no association was observed for MCS. Increasing each specific animal source’s food intake was associated with better PCS after adjusting for all covariates. However, for MCS, positive associations were only observed in seafood consumption among men and eggs among women. Restricted cubic splines showed a substantial dose-response association between intake frequency of animal-source foods and PCS, both in men and women. The study suggests that a diverse intake of animal-sourced foods can potentially improve the HRQoL of Chinese adults.

## 1. Introduction

Health-related quality of life (HRQoL) is a multidimensional concept that encompasses perceptions of overall self-reported health as well as physical, psychological, social, and role functioning [1]. The question of how to enhance people’s health-related quality of life has grown in importance as a focus of public health research and practice due to the aging population and increased life expectancy [2]. While there are numerous factors that can influence HRQoL, diet—a modifiable lifestyle factor—has been identified as one of the most significant determinants of health status, along with financial status and changes in living conditions [3,4].

Current cross-sectional and cohort studies appear to have observed that certain dietary patterns are associated with better self-rated health and HRQoL across various domains, such as the Mediterranean diet, which was significantly associated with improvement in at least one of the HRQoL domains [5]. An eight year follow-up study in the U.S. found that an increase in the healthy plant-based diet index was significantly associated with enhanced physical HRQoL in older women and improved mental HRQoL in younger women [6]. A positive association between adherence to the Mediterranean diet and HRQoL seemed to also be established [7,8]. Unfortunately, the evidence regarding specific foods or food groups on HRQL is still limited, and the results are not consistent across different populations [9,10,11,12].

Animal-sourced foods provide high-quality proteins and micronutrients that play a distinct role in the psychological and physical health of men and women [13], which are difficult to obtain from plant-sourced foods alone [14]. Especially in the elderly, it was moderately established that appropriately increasing the intake and diversity of animal protein based on the recommended intake for adults was an effective measure to avoid the risk of malnutrition [15]. Diets vary significantly across ethnic, cultural, and geographical backgrounds. The Western diet, which is thought to be characterized by a high-fat meat intake, is believed to be related to the occurrence of various diseases, especially cardiovascular diseases [5]. In contrast, the Chinese tend to consume more vegetables and staple foods but fewer animal products. Given the different dietary patterns, there appeared to be differences in metabolic and genetic responses between Asian and Western populations [16,17]. What is more, women probably eat less meat than men generally, especially beef and pork [18]. Such differences in the consumption of dietary food should be taken into account when interpreting the association between diet and the health status of a given population, and the contribution of animal food intake to diet quality and health requires further assessment [19]. Previous studies on the relationship between meat and HRQoL are still limited, and the conclusions are not uniform, especially in Chinese women and mental health [20].

The assessment of dietary diversity serves as a rapid, user-friendly, and key dimension for assessing diet quality [21]. Higher dietary diversity has been associated with favorable nutrient composition [22], an increased likelihood of adequate nutrient intake [23], elevated concentrations of antioxidant blood markers [24], lower cardiovascular and non-alcoholic fatty liver disease risk factors [25,26], and metabolic syndrome [27], which suggests that higher diet diversity could improve the health status of the population. The association of life quality with dietary diversity has not been addressed sufficiently. As a result, this study evaluated the dietary diversity of animal foods and further examined whether increasing the diet diversity of animal-source foods improved the HRQoL of Chinese using large-scale data.

## 2. Materials and Methods

### 2.1. Study Design and Participants

This study used baseline data from the Regional Ethnic Cohort Study in northwest China (RECS), and the detailed study design has been described elsewhere [28]. Briefly, participants were recruited between June 2018 and May 2019 from northwestern China, including all five provinces (Shaanxi, Xinjiang, Ningxia, Gansu, and Qinghai). A face-to-face questionnaire interview was conducted to collect baseline information on participants’ demographic and socioeconomic characteristics, lifestyle factors, environmental exposures, medical history, mental health status, and reproductive history. The present study included participants from the Shaanxi cohort (n = 48,025). Participants aged < 18 years or > 80 years (n = 138) were excluded. Additionally, the participants who had missing data for HRQoL (n = 2204) and incomplete dietary data (n = 5686) were excluded, resulting in a total of 39,997 participants in the final analyses (Figure S1). Compared to those we excluded, the groups we included tended to be more women, older people, people with lower socioeconomic status (SES), rural residents, less likely to smoke or drink alcohol, engage in physical activity, and have less healthy eating habits (Table S1). This study was conducted according to the guidelines laid down in the Declaration of Helsinki, and all procedures involving all participants were approved by the Human Research Ethics Committee of the Xi’an Jiaotong University Health Science Center (No. XJTU2016-411). Written informed consent was obtained from all participants.

### 2.2. Dietary Assessment and Animal-Source Food Diet Diversity

Diet information was collected using a semi-quantitative food frequency questionnaire (FFQ) with a list of 31 food groups. It is an adapted version used in the China Kadoorie Biobank, which was shown to have good validity and reproducibility [29]. Participants were asked to report the frequency and portion size of each food group consumed according to their dietary habits over the past 12 months, and one of five options for each food group in this FFQ was asked to select “daily”, “4–6 times/week”, “1–3 times/week”, “1–3 times/month” and “none or rarely”. In the data analysis, they were quantified at 7, 5, 2, 0.5, and 0 times a week, respectively. According to the Chinese recommended daily intake for adults [30], pork, mutton, beef, poultry, seafood, eggs, pure milk, and yogurt were selected as animal source foods, which were used to establish the animal source food diet diversity score (ASFDDS) in the present study [14]. In our analysis, we further combined pork, mutton, and beef into red meat, and poultry was regarded as white meat. For each animal source food item, if the reported intake frequency was equal to or greater than once per day, it received a score of 1; otherwise, a score of 0 was assigned. The ASFDDS ranged from 0 to 8 points; higher scores indicated greater diet diversity in terms of consumption of different types of animal-sourced foods. Furthermore, the participants were divided into two groups based on their ASFDDS scores: the high-diversity group (ASFDDS ≥ 1) and the low-diversity group (ASFDDS = 0).

### 2.3. Health-Related Quality of Life

The 12-Item Short Form Survey (SF-12), a shortened version of the SF-36, was used to assess HRQoL [31]. This study used the Chinese standard version of the SF-12 scale, which has been demonstrated to be valid for Chinese populations with good reliability and validity [32,33]. Additionally, the questions from SF-12 were read to the participants, who were then asked to provide a rating. It consists of various domains that include physical function, role physical (limitations due to physical health problems), bodily pain, general health—collectively forming the Physical Component Score (PCS), as well as vitality, social functioning, role emotional (limitations due to emotional problems), and mental health—collectively forming the Mental Component Score (MCS). PCS/MCS scores range from 0 to 100, with higher values indicating better HRQoL [34]. For data analysis in the present study, a strength-based approach was used. This involved establishing the hypothesis that increasing the diet diversity of animal-source foods could further improve participants’ HRQoL. Consequently, better PCS/MCS scores were defined as those exceeding the 90th percentile amongst all participant scores.

### 2.4. Covariates

Gender, age, SES, marital status, smoking, drinking, physical activity, history of chronic disease, sleeping problems, unhealthy eating habits, staple food intake, other diet diversity score (ODDS), and body mass index (BMI) were included as covariates in this study [35,36,37]. SES was calculated using a comprehensive index that considered family income, education level, and occupation. It was then divided into tertiles (high, medium, and low). Marital status was defined as either married or unmarried. Smoking was defined as smoking on a daily or weekly basis. Drinking was classified as at least once a month or once a week. Metabolic equivalents [38] were used to assess physical activity. A history of chronic disease refers to the presence of medically diagnosed non-communicable diseases, including hypertension, diabetes, and pulmonary heart disease. Sleeping problems were characterized by taking more than half an hour to fall asleep, waking up early in the morning with difficulty falling back asleep, taking sleeping pills at least once a day for assistance, and poor sleep quality resulting in difficulties staying awake during the day. Unhealthy eating habits included behaviors related to snacking convenience foods, midnight snacks, bacon-processed foods, fried foods, and fast-food consumption. Additionally, it entailed habitually skipping breakfast and frequently eating out. These behaviors were deemed unhealthy due to their potential psychological and physical consequences [39]. Due to the questionnaire’s limitations, we evaluated energy intake using other diet diversity scores (ODDS), including staple food intake but not animal foods. The accumulated intake of rice, cooked wheat food, and cereals was designated as staple food intake. According to the Chinese recommended daily intake for adults [30], ODDS was calculated for the six major food groups: staple food, white tubers and roots, vegetables, fruits, legumes/nuts/seeds, and sweets. One ODDS unit represented at least five times a week the consumption of any food group without considering a minimum intake. Cereals and oil were excluded from ODDS construction as they are consumed daily by most Chinese individuals [40]. Food intake frequencies were translated into times per week; for example, 4–6 times per week equaled 5 times per week. Height, waist circumference, and weight were measured by trained staff members. BMI was calculated as weight (kg) divided by height squared (m^2^).

Based on currently available professional knowledge, a directed acyclic graph (DAG) was used to determine the causal relationship among the 10 confounding variables [41]. Finally, to select the smallest set of adjustment covariates, smoking, drinking, ODDS, staple foods, and sleeping problems were excluded because these variables lie on the causal pathway between the other variables and HRQoL. In the adjusting analysis (Figure S2), the remaining covariates were included. Missing data for these aforementioned variables was less than 5%, and various imputation techniques were used to handle missing data for subsequent statistical analyses.

### 2.5. Statistical Analyses

Continuous variables, presented as mean and deviation, were analyzed by ANOVA, while categorical variables, presented as frequencies and percentages, were analyzed by χ^2^. All variables were presented in the total sample and separately by gender. Logistic regression models were used to estimate odds ratios and 95% CI for associations of PCS and MCS (as dependent variables) with ASFDDS or intake of specific animal source foods (as independent variables). In line with a strength-based approach to data analysis, which hypothesized that increasing the diet diversity of animal-source foods could improve HRQoL, as a result, odds ratios greater than 1 indicated a better HRQoL status. With the adjustment of covariates based on a priori defined DAG, a series of adjusted models was established to control the impact of potential covariates. Model 1 was a crude model without adjusting for any covariates. Model 2 was adjusted for age and gender (in total participants). Model 3 was additionally adjusted for SES, marital status, residence, physical activity, history of chronic disease, personal eating habits, and BMI. Given the possibility of gender differences in the relationship between diet and HRQoL (23,36), all analyses were conducted on the entire sample as well as separately on men and women. Using restricted cubic splines, the dose–response association was assessed between HRQoL and log-transformed total frequency of ASF intake, which was used as an alternative way to present ASF diversity, with trimmed observations at 1% and 99% of the distribution [42]. Knots were placed at the 10th, 50th, and 90th percentiles of the exposure distribution [43].

Additional analyses were performed. Firstly, subgroup analyses were conducted to explore the heterogeneity of the association between ASFDDS and HRQoL, in which the multiplication model was used to identify the interaction effect. Secondly, to minimize the chances of reverse causation, e-values associated with the optimal dose were calculated, estimating the plausibility of bias from unmeasured confounding. Lastly, to verify the robustness of ASFDDS associated with HRQoL, several sensitivity analyses were conducted with repeating regression analysis (a) in the participants without nutrition-related diseases (hypertension, diabetes, and pulmonary heart disease), (b) with adjustment for all potential covariates including drinking, smoking, ODDS, staple food, and sleeping problem-based modal 3, (c) in the unimputed data, and (d) by sequential exclusion of each food from the ASFDDS.

Statistical analyses were performed using SPSS 26.0 and R 4.0 software. All tests conducted were two-sided, with *p* < 0.05 indicating statistical significance.

## 3. Results

### 3.1. Characteristics of the Participants

Among the 39,997 participants, 64.0% were women, and the average age was 50.43 ± 13.09. Table 1 shows the main characteristics and food intake of participants stratified by gender. The mean PCS was 49.86 in men and 49.2 in women, and the mean MCS was 52.7 and 52.34 in women. In the current study, men were more likely to have high SES, more physical activity, a large BMI, and to be smokers and drinkers than women. Additionally, men had more staple food intake and unhealthy eating habits but fewer ODDS. The average animal source of food intake was 5.52 ± 5.54 times/week in men and 3.82 ± 4.74 times/week in women. The average ASFDDS was 1.07 ± 1.24 and 0.92 ± 1.17, respectively. Men consumed more pork, mutton, beef, poultry, seafood, eggs, and pure milk but less yogurt than women.

### 3.2. The Association of HRQoL with ASFDDS

Table 2 shows the overall and gender-specific associations between ASFDDS and PCS/MCS. In total participants, the greater ASFDDS (per 1 point increase in the score) was associated with increased odds of better PCS (OR = 1.09, 1.04–1.14; *p* < 0.001) in the full adjusted model. Compared with those with an ASFDDS score of 0, the participants with higher ASFDDS (≥2) had increased odds of better PCS (OR = 1.26, 1.13–1.40; *p* < 0.001) in the full adjusted model. However, there was no conclusive evidence of an association between ASFDDS and MCS.

The results indicated some differences between men and women. In men, greater ASFDDS (per 1 point increase in the score) was associated with increased odds of both better PCS (OR = 1.08, 1.01–1.15; *p* = 0.026) and better MCS (OR = 1.11, 1.04–1.20; *p* = 0.003) in the full adjusted model. Compared with those with an ASFDDS score of 0, the participants with higher ASFDDS (≥2) had increased odds of both better PCS (OR = 1.16, 1.01–1.34; *p* = 0.002) and better MCS (OR = 1.24, 1.03–1.48; *p* = 0.020) in the full adjusted model. In women, the greater ASFDDS (per 1 point increase in the score) was associated with increased odds of better PCS (OR = 1.10, 1.04–1.16; *p* < 0.001) in the full adjusted model. Compared with those with an ASFDDS score of 0, the odd ratios for better PCS were 1.15 (1.01–1.29; *p* = 0.017) for the participants with ASFDDS = 1 and 1.34 (1.16–1.54; *p* < 0.001) for participants with ASFDDS ≥ 2. However, no significant association was found between ASFDDS and MCS.

### 3.3. The Association of HRQoL with Intake of Specific Animal Source Food

Table 3 shows associations of PCS/MCS with specific, overall animal source food intake after adjusting for all covariates by gender. No matter whether intake of specific or overall animal source food was associated with increased odds of better PCS in both sexes, the significant association between intake of eggs and PCS was not observed only in men. However, no consistent, significant relationship was found between ASFDDS and MCS. Only intake of seafood was associated with increased odds of better MCS in total participants and men, while intake of eggs was associated with increased odds of better MCS in total participants and women.

**Figure 1:**
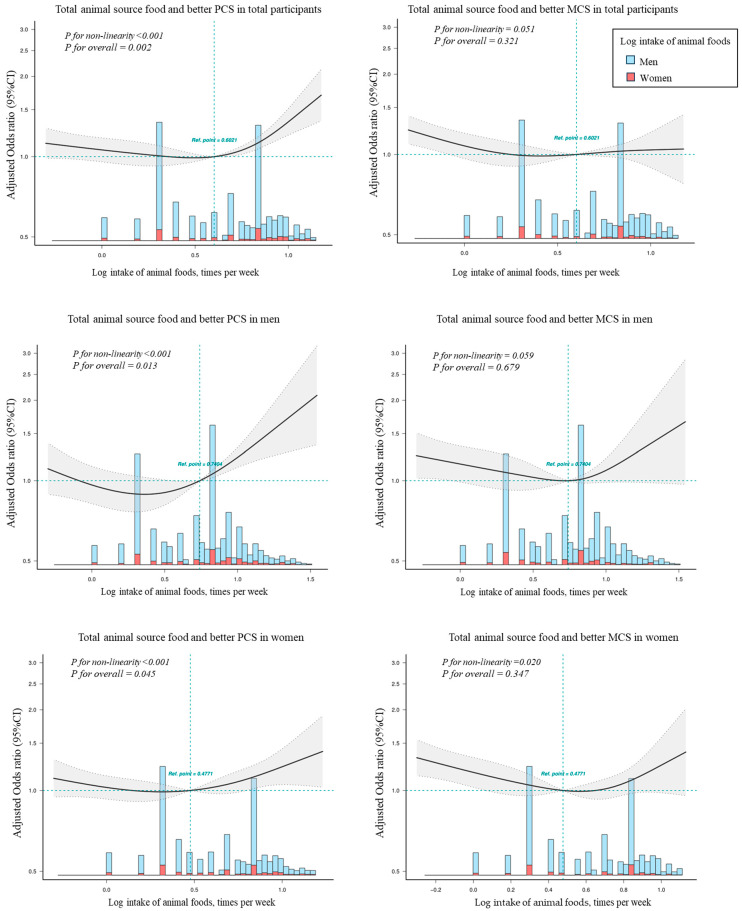
Dose–response association between intake of animal source food and physical component score (PCS) and mental component score (MCS). Dose–response associations were assessed with restricted cubic splines with knots at the 10th, 50th, and 90th centiles of the distribution of the log-transformed animal source of food. Odds ratios were adjusted for age, sex (in total analyses), SES, marital status, residence, physical activity, BMI, history of chronic disease, and unhealthy eating habits. Shaded areas represent 95% Cis.

Figure 1 displays the results from restricted splines analysis with multivariable adjustment about dose–response between total animal source food consumption and PCS/MCS. In total participants, a significantly higher PCS was observed when they consumed more intake of animal source food (i.e., the median log-transformed total score of animal source food was 0.60, meaning animal source food intake was more than four times per week). No significant non-linear association was detected for total animal-source food consumption and MCS (*p* > 0.05). In men, a higher PCS was observed with a higher intake of animal source food. There was an increasing trend in the odds of better MCS when the frequency of animal-sourced food intake was beyond 5.5 times per week (median), but no significant non-linear association was detected for that (*p* > 0.05). In women, there is a flat phase before the median intake of animal-source food (three times per week) and then an increase in odds of better PCS appeared. The association with MCS and animal source food presented an approximate U-shape.

### 3.4. Subgroup Analyses and Sensitivity Analyses

Subgroup analyses indicated that a positive association between higher ASFDDS and better PCS was found in all subgroups after adjusting for potential covariates. However, there was some significant heterogeneity found in such subgroups as age, SES, chronic diseases, and unhealthy food intake. In particular, higher ASFDDS seemed to have a greater impact on older participants. A similar result was observed for MCS, and greater heterogeneity was found in the age, sex, and SES subgroups (Figure S3). The e-value for the main results is presented in Table S2. The e-values about odds ratios between ASFDDS and PCS were 1.60 in men and 2.01 in women. That meant that residual confounding could explain the observed association between PCS and ASFDDS in women if there exists an unmeasured covariate with a relative risk of at least as large as 2.01. In the present study, the ORs for some potential factors were 1.13 for SES and 1.19 for marriage statue. It was not likely that an unmeasured or unknown confounder would have a substantially greater effect on HRQoL than these known risk factors.

When restricting the analysis to participants with hypertension, diabetes, and pulmonary heart disease (Table S3), adjusting further for drinking, smoking, ODDS, staple food, and sleeping problems (Table S4), or using the raw data without imputing (Table S5), the association of interest did not change largely. Figure S4 showed that after sequentially excluding each individual food used for establishing ASFDDS, the association of interest was consistent with the primary analyses, which suggested a robust positive association between ASFDDS and quality of life.

## 4. Discussion

This large-sample study of Chinese adults aged 20 to 80 years contributes to our understanding of the links between health-related quality of life and the diet diversity of animal-source foods, which is important for public health. We discovered a positive correlation between ASFDDS and better PCS, with a greater impact on women. Compared to those with low meat intake, male participants with ASFDDS greater than or equal to two had a 16.4% increased likelihood of better PCS, while women had a 33.7% increase. For MCS, the odd ratios for better MCS increased by 23.9% in men with ASFDDS greater than or equal to two compared with those with ASFDDS = 0, but no such association was found in women. Moreover, regardless of gender, specific animal-source foods were found to be positively associated with better PCS. Increased seafood consumption was associated with better MCS in men, while eggs were associated with better MCS in women. ASF and PCS showed a dose-response relationship in both men and women, with the increasing trend being more pronounced when ASF intake exceeded the median.

Dietary nutrition plays an important role as a modifiable factor for health outcomes. Previous evidence from various studies on dietary nutrition and quality of life was primarily focused on developed countries but yielded inconsistent conclusions. A study in North America showed that red meat, pastries, and fast food-based Western dietary patterns have a negative impact on quality of life [44]. The inconsistency could be attributed to differences among populations. However, the present study revealed a positive association between animal food and better HRQoL, particularly for better PCS, which partly differed from previous studies. A reasonable explanation could be that the dietary pattern in China changed rapidly during the past two decades along with economic development, but the intake of animal protein in China was still lower than the recommended amount of the Chinese dietary guidelines and much lower than that in developed countries such as the US [45]. Therefore, Western-based dietary research results may not be directly applicable to the Chinese population due to differences in culture and dietary patterns. In our study, animal food intake was limited, especially among women, with nearly two-thirds of participants consuming little or no animal food per day. Additionally, the diversity of animal food intake was very low in both men and women. These factors may partly account for the significantly positive association between animal food and HRQoL in the present study. Consequently, our findings suggest that the intake of animal food sources and their diversity should be appropriately increased to effectively improve health-related quality of life in Chinese adults.

The impact of the diet diversity of animal-source foods on HRQoL seemed to be stronger in some subgroups. Firstly, a stronger association between the ASFDDS and HRQoL was found in the participants with lower SES. This result supports the widely held belief that the close relationship between diet cost and diet quality may be a contributing factor to the observed socioeconomic disparities in diet and health [46]. Improving the diversity of animal-source food consumption may be a cost-effective public health intervention to effectively improve the psychological and physiological health of the Chinese population [47]. Secondly, we found that the diet diversity of animal-source foods had a greater impact on PCS in women. Women consumed less pork, mutton, beef, poultry, seafood, eggs, and pure milk than men, except for yogurt, which might explain this gender difference. Low SES may reflect a woman’s lower accessibility to food types of choice. Iron deficiency related to poor dietary nutrition is problematic for pregnant women and infants living in the U.S. and Europe, particularly in lower socioeconomic groups [48]. Low SES may also reflect a woman’s lower education level, which could influence her diet and that of her family [49]. Meanwhile, lower education and economic status of women were important factors causing the health disparity between both genders, in which dietary intake was an important mediator [50]. Given these heterogeneous results, it is suggested that more attention should be paid to women with low SES. Thirdly, the present study showed that egg consumption was associated with both PCS and MCS among women but not men, potentially because of different physiological mechanism variations between men and women. For instance, a sex difference in glucose metabolism has been described by many studies, which found that women tend to have greater insulin sensitivity than men [51]; moreover, the occurrence of insulin resistance seems to be higher in men [52]. Egg consumption was associated with a decreased risk of dyslipidemia among Chinese women but not men, and a higher intake level is associated with a lower prevalence of metabolic syndrome, particularly among females [53]. These studies suggested a significant gender difference in dietary nutrition in the Chinese population, which aligns with our findings [54]. In addition, mounting evidence has demonstrated that fish intake may reduce the risk of cognitive function. A Chinese study presented that fish or aquatic products may reduce mental stress by improving psychological health via antioxidant action and inflammatory responses; this protective effect is more pronounced in men [55], consistent with our findings indicating that proper intake of fish was beneficial to mental health in men.

Although a positive association was observed between the quality of life and animal source food, the mechanisms involved remain unclear, and several potential mechanisms are presented here. First, animal foods such as eggs, milk, and meat are the main sources of high-quality protein and essential amino acids; the proportion of nitrogenous acids is close to that of the human body and is easily digested and absorbed by the human body [56]. Second, animal foods provide a variety of micronutrients, such as fat-soluble vitamins, vitamin B, and minerals, that other foods might lack [57]. It is worth noting that traditional rice-based diets with low micronutrient density have a negative impact on energy, calcium, iron, and zinc status [58], and these intakes have been linked to depression [59]. Vitamin B-12 status can have an impact on the nervous system, gastrointestinal system, and overall energy levels [60], and vitamin D can help reduce the risk of falls, fractures, and osteoporosis by lowering parathyroid hormone levels and playing an important role in neuroendocrine functioning in the brain [61]. Finally, animal foods may be linked to cognitive impairment and brain atrophy by affecting muscle mass and strength [62]. Additionally, prior studies have revealed that vegetarians and vegans are significantly more likely to experience depression, anxiety, and/or self-harm [63].

Men and women may differ physiologically and biochemically, which could explain gender differences in the association between ASF intake and both physical and mental health. There was evidence that age-related declines in estrogen levels in women appeared to lessen bone and muscle tissue more than in men, increasing the risk of weakness and, ultimately, the occurrence of physical diseases [64], which partly explains why meat consumption was better for women’s PCS. However, the underlying mechanisms of diet and MCS could be complicated and remain unclear. Women are twice as likely as men to experience an anxiety disorder [65], suggesting that psychological problems may be more complex among the female population. Furthermore, different dietary patterns could partly account for the gender-specific association of MCS with ASF. Studies found that inadequate intake of branched-chain amino acids and micronutrients like zinc harmed mental health [59,66]. In the present study, women consume less meat than men, and it is unrealistic to substantially increase women’s diversity of animal foods. Therefore, the underlying reasons for the gender-modification effect still warrant further investigation.

### Strengths and Limitations

The strength of the present study lies in the large sample size and wide age range. The majority of earlier studies concentrated on how dietary habits affected elderly people’s HRQoL, while the present study covered a wider age span and included more middle-aged adults. A series of sensitivity analyses were also conducted to confirm the robustness of this positive association. Nevertheless, several limitations of this study need to be acknowledged. First, the cross-sectional design used in this study did not allow the establishment of an appropriate temporal sequence to demonstrate causality because reverse causation cannot be ruled out. However, the relatively large e-values showed that this possibility is relatively minimal. Second, the role of diet in promoting health and preventing disease is difficult to elucidate clearly because of the complexity of foods in dietary nutrition. However, a recent meta-analysis including 30 studies showed that diet variety had a small-to-medium effect on dietary energy and nutrient intake [67]. Meanwhile, we added the intake of staple foods and personal dietary preferences to the analysis model to reduce the influence of energy intake and eating habits on the results to some extent. Moreover, in the present study, we could not distinguish processed animal foods due to the limited dietary information in the questionnaires. Third, although we adjusted for numerous potential confounders, residual confounding may not be completely rooted. Fourth, although a priori defined directed acyclic graphs were used to identify key factors known to influence the causal associations, residual or unmeasured confounding may still be present. Lastly, a large proportion of women and those with low socioeconomic status were included in this study. It needs to be very cautious when applying our results to other nationalities due to the limitations of population characteristics and the fact that generalizability may be limited. However, the results of this study could probably be of some reference to other nationalities or populations with lower socioeconomic status and similar dietary features to our participants, like low availability and diversity of animal foods.

## 5. Conclusions

Increasing diet diversity of animal source food and intake frequency of specific animal source food are both positively associated with improved physical components in Chinese, especially in women. Interestingly, men show a positive impact of increasing diversity of animal-source foods on mental component improvement. These findings suggest that tailored dietary interventions focusing on improving animal-source food diversity could enhance the quality of life for the Chinese population. However, further research is needed to validate these findings and establish causality.

## Figures and Tables

**Table 1:** The characteristics of the participants by gender ^1^.

	Men n = 14,383	Women n = 25,614	Total n = 39,997	*p*-Value
Age (year), $\bar{x}\pm s$	50.28 ±13.97	50.53 ±12.56	50.43 ±13.09	0.050
Age group, n (%)				0.041
≤40	3946 (27.4)	5588 (21.8)	9534 (23.8)	
41–50	2397 (16.7)	5259 (20.5)	7656 (19.1)	
51–60	3679 (25.6)	8216 (32.1)	11,895 (29.7)	
≥61	4361 (30.3)	6551 (25.6)	10,912 (27.3)	
SES, n (%)				<0.001
Low	4130 (28.7)	11,402 (44.5)	15,532 (38.8)	
Median	3996 (27.8)	7490 (29.2)	11,486 (28.7)	
High	6257 (43.5)	6722 (26.2)	12,979 (32.4)	
Married, n (%)	12,469 (86.7)	22,831 (89.1)	35,300 (88.3)	<0.001
Urban, n (%)	5647 (39.3)	6374 (24.9)	12,021 (30.1)	<0.001
Drinking, n (%)	2785 (19.4)	454 (1.8)	3239 (8.1)	<0.001
Smoking, n (%)	5897 (41.0)	121 (4.7)	6018 (15.1)	<0.001
BMI (kg/m^2^), $\bar{x}\pm s$	24.24 ±3.45	23.57 ±3.62	23.82 ±3.57	<0.001
BMI group, n (%)				<0.001
<18.5	520 (3.6)	1334 (5.2)	1854 (4.6)	
18.5–23.9	6452 (44.9)	13,544 (52.9)	19,996 (50.0)	
≧24.0	7411 (51.5)	10,736 (41.9)	18,147 (45.4)	
History of chronic disease, n (%)	5413 (37.6)	9288 (36.3)	14,701 (36.8)	0.029
Physical activity (MET:h/d), $\bar{x}\pm s$	25.48 ±13.61	20.01 ±11.72	21.94 ±12.69	<0.001
More than 21.4 MET, n (%)	8868 (61.7)	11,131 (43.5)	19,999 (50.0)	
Frequency of staple food intake (times/week), $\bar{x}\pm s$	11.62 ±4.48	11.56 ±4.52	11.57 ±4.52	0.227
More than 11 times/week, n (%)	6517 (45.3)	11,333 (44.2)	17,850 (44.6)	<0.001
Unhealthy eating habits(times/week), $\bar{x}\pm s$	4.85 ±6.94	3.54 ±5.80	4.01 ±6.27	<0.001
More than three times/week, n (%)	7812 (54.3)	11,320 (44.2)	19,132 (47.8)	<0.001
ODDS, $\bar{x}\pm s$	1.95 ±0.95	2.02 ±0.92	1.99 ±0.93	<0.001
Intake of animal source foods (times/week), $\bar{x}\pm s$	5.52 ±5.54	3.82 ±4.74	4.43 ±5.11	<0.001
Pork, $\bar{x}\pm s$	3.12 ±2.65	2.32 ±2.55	2.61 ±2.62	<0.001
Mutton, $\bar{x}\pm s$	0.57 ±1.25	0.31 ±0.97	0.41 ±1.08	<0.001
Beef, $\bar{x}\pm s$	0.68 ±1.36	0.40 ±1.07	0.50 ±1.19	<0.001
Poultry, $\bar{x}\pm s$	0.71 ±1.40	0.47 ±1.16	0.56 ±1.26	<0.001
Seafood, $\bar{x}\pm s$	0.44 ±1.01	0.32 ±0.89	0.36 ±0.94	<0.001
Eggs, $\bar{x}\pm s$	2.57 ±2.61	2.26 ±2.57	2.37 ±2.59	<0.001
Pure milk, $\bar{x}\pm s$	1.56 ±2.43	1.39 ±2.43	1.45 ±2.43	<0.001
Yogurt, $\bar{x}\pm s$	0.91 ±1.75	0.93 ±1.87	0.92 ±1.83	0.512
ASFDDS, $\bar{x}\pm s$	1.07 ±1.24	0.92 ±1.17	0.98 ±1.20	<0.001
0, n (%)	5570 (38.7)	11,875 (46.4)	17,445 (43.6)	
1, n (%)	5084 (35.3)	7938 (31.0)	13,022 (32.6)	
2, n (%)	2199 (15.3)	3353 (13.1)	5552 (13.9)	
>2, n (%)	1530 (10.6)	2448 (9.6)	3978 (9.9)	
Life quality score				
PCS, $\bar{x}\pm s$	49.86 ±7.20	49.29 ±7.44	49.49 ±7.35	<0.001
Better PCS	1643 (11.4)	2745 (10.7)	4388 (11.0)	0.016
MCS, $\bar{x}\pm s$	52.70 ±6.98	52.34 ±7.18	52.47 ±7.11	<0.001
Better MCS	1588 (11.0)	2676 (10.4)	4264 (10.7)	0.034

SES socioeconomic status, BMI body mass index, ODDS other diet diversity score, ASFDDS animal source food diet diversity score, PCS physical component score, MCS mental component score, IQR interquartile range. *p*-value for the chi-square or ANOVA test between different genders. ^1^ Data are mean ± SD, n (%) unless otherwise indicated.

**Table 2:** Association of PCS and MCS with ASFDDS ^1^.

		ASFDDS		ASFDDS Category				
				0 (n = 17,445)	1 (n = 13,022)		≥2 (n = 9530)	
		OR (95%CI)	*p*	Reference	OR (95%CI)	*p*	OR (95%CI)	*p*
Total								
PCS	Model 1	1.12 (1.10, 1.14)	<0.001	Ref.	1.00 (0.93, 1.08)	0.959	1.33 (1.23, 1.43)	<0.001
	Model 2	1.04 (1.02, 1.07)	0.001	Ref.	0.93 (0.86, 1.00)	0.063	1.07 (0.99, 1.17)	0.091
	Model 3	1.09 (1.04, 1.14)	<0.001	Ref.	1.06 (0.97, 1.17)	0.179	1.26 (1.13, 1.40)	<0.001
MCS	Model 1	0.91 (0.89, 0.94)	<0.001	Ref.	0.85 (0.79, 0.91)	<0.001	0.75 (0.69, 0.82)	<0.001
	Model 2	1.00 (0.97, 1.03)	0.899	Ref.	0.91 (0.85, 0.98)	0.012	0.97 (0.89, 1.06)	0.496
	Model 3	1.03 (0.98, 1.07)	0.252	Ref.	0.97 (0.89, 1.06)	0.469	1.12 (1.00, 1.26)	0.056
Men								
PCS	Model 1	1.10 (1.06, 1.15)	<0.001	Ref.	0.89 (0.78, 1.00)	0.059	1.25 (1.10, 1.41)	0.001
	Model 2	1.04 (1.00, 1.08)	0.062	Ref.	0.83 (0.73, 0.94)	0.003	1.04 (0.91, 1.18)	0.609
	Model 3	1.08 (1.01, 1.15)	0.026	Ref.	0.93 (0.80, 1.08)	0.931	1.16 (1.01, 1.34)	0.002
MCS	Model 1	0.93 (0.89, 0.98)	0.002	Ref.	0.84 (0.74, 0.95)	0.004	0.80 (0.70, 0.92)	0.001
	Model 2	1.03 (0.99, 1.08)	0.157	Ref.	0.93 (0.82, 1.14)	0.207	1.07 (0.93, 1.23)	0.350
	Model 3	1.11 (1.04, 1.19)	0.003	Ref.	1.02 (0.89, 1.18)	0.759	1.24 (1.03, 1.48)	0.020
Women								
PCS	Model 1	1.12 (1.09, 1.16)	<0.001	Ref.	1.07 (0.97, 1.17)	0.173	1.36 (1.23, 1.50)	<0.001
	Model 2	1.05 (1.01, 1.08)	0.010	Ref.	1.00 (0.91, 1.10)	0.949	1.09 (0.98, 1.21)	0.120
	Model 3	1.10 (1.04, 1.16)	0.001	Ref.	1.15 (1.01, 1.29)	0.017	1.34 (1.16, 1.54)	<0.001
MCS	Model 1	0.89 (0.86, 0.93)	<0.001	Ref.	0.85 (0.77, 0.93)	<0.001	0.72 (0.64, 0.80)	<0.001
	Model 2	0.97 (0.94, 1.01)	0.188	Ref.	0.90 (0.82, 0.99)	0.031	0.91 (0.81, 1.02)	0.097
	Model 3	0.97 (0.93, 1.04)	0.973	Ref.	0.94 (0.84, 1.05)	0.260	1.04 (0.90, 1.22)	0.580

PCS physical component score, MCS mental component score, OR odds ratio, CI confidence interval. Model 1 was a crude model. Model 2 was adjusted for age [and gender (in total analyses)]. Model 3 was adjusted for the variables adjusted for in Model 2 and SES: marital status, residence, physical activity, history of chronic disease, unhealthy eating habits, and BMI. ^1^ The odds ratio greater than 1 indicated a better status for PCS/MCS.

**Table 3:** Association of PCS and MCS with the intake frequency of specific and overall animal sources of food ^1^.

	PCS		MCS	
	OR (95% CI)	*p*	OR (95% CI)	*p*
Red meat (pork, mutton, beef)				
Total	1.02 (1.01, 1.03)	0.003	1.00 (0.99, 1.02)	0.509
Men	1.02 (1.00, 1.04)	0.017	1.01 (0.99, 1.03)	0.418
Women	1.02 (1.00, 1.03)	0.005	1.00 (0.98, 1.02)	0.908
White meat (poultry)				
Total	1.08 (1.04, 1.11)	<0.001	0.97 (0.93, 1.02)	0.239
Men	1.07 (1.04, 1.11)	<0.001	1.02 (0.97, 1.06)	0.451
Women	1.07 (1.04, 1.10)	<0.001	0.99 (0.95, 1.04)	0.706
Seafood				
Total	1.13 (1.08, 1.18)	<0.001	1.06 (1.00, 1.12)	0.050
Men	1.13 (1.06, 1.20)	<0.001	1.09 (1.01, 1.18)	0.032
Women	1.13 (1.07, 1.20)	<0.001	1.03 (0.95, 1.11)	0.479
Eggs				
Total	1.02 (1.01, 1.04)	0.006	1.02 (1.01, 1.04)	0.009
Men	1.01 (0.98, 1.04)	0.463	1.02 (1.00, 1.05)	0.115
Women	1.03 (1.01, 1.05)	0.004	1.02 (1.00, 1.04)	0.041
Dairy				
Total	1.04 (1.02, 1.06)	<0.001	1.00 (0.98, 1.02)	0.979
Men	1.04 (1.01, 1.07)	0.005	1.01 (0.99, 1.04)	0.363
Women	1.04 (1.02, 1.06)	<0.001	0.99 (0.97, 1.02)	0.484
Overall animal source of food				
Total	1.01 (1.01, 1.03)	<0.001	1.00 (0.99, 1.01)	0.528
Men	1.02 (1.01, 1.04)	<0.001	1.02 (0.99, 1.05)	0.115
Women	1.02 (1.01, 1.03)	0.001	1.00 (0.99, 1.01)	0.976

PCS physical component score, MCS mental component score, OR odds ratio, CI confidence interval. ^1^ Adjusted for age, sex (in total analyses), SES, marital status, residence, physical activity, history of chronic disease, unhealthy eating habits, and BMI.

## Data Availability

The datasets are not available for download to protect the confidentiality of the participants. The data are held at the School of Public Health, Xi’an Jiaotong University Health Science Center.

## Supplementary Materials

The following supporting information can be downloaded at: https://www.mdpi.com/article/10.3390/nu15194183/s1, Table S1: The characteristics of the participants included and excluded; Table S2: The e-value of the association between ASFDDS and PCS; Table S3: The association of physical component score (PCS) and mental component score (MCS) with animal source food diet diversity score (ASFDDS) based on logistic regression models (excluding participants with hypertension, diabetes, and pulmonary heart disease, n = 25,296); Table S4: The association of physical component score (PCS) and mental component score (MCS) with animal source food diet diversity score (ASFDDS) based on logistic regression models with all covariates without selection (adding drinking, smoking, ODDS, staple food, and sleeping problems in model 3); Table S5: The association of physical component score (PCS) and mental component score (MCS) with animal source food diet diversity score (ASFDDS) based on logistic regression models with unfilled data (n = 35,783); Figure S1: The flow chart for study participant selection; Figure S2: A priori defined directed acyclic graph; Figure S3: Subgroup analyses for physical component score (PCS), mental component score (MCS), and animal source food diet diversity score (ASFDDS); Figure S4: The association between the sequential exclusion of each food from the ASFDDDS and PCS/MCS.

## Abbreviations

ASF: Animal source food; ASFDDS: Animal source food diet diversity score; BMI: Body mass index; DAG: Directed acyclic graph; FFQ: Food frequency questionnaire; HRQoL: Health-related quality of life; IQR Interquartile range; MCS: Mental component score; ODDS: Other diet diversity score; PCS: Physical component score; SES: Socioeconomic status; SF-12: 12-Item Short Form Survey.
