# Supplementary material for: Is Increasing Diet Diversity of Animal-Source Foods Related to Better Health-Related Quality of Life among Chinese Men and Women?
Source: Nutrients. 2023 Sep 27;15(19):4183. doi: 10.3390/nu15194183 (PMC10574670; doi:10.3390/nu15194183)
Supplement: Supplementary file 1 [file nutrients-15-04183-s001.zip › nutrients-2594406-supplementary.pdf]

Supplemental documents

Is increasing diet diversity of animal-source foods related to better health-related quality of life among Chinese men and women?

Hui Jing, et al.

Contents

Table S1. The characteristics of the participants included and excluded

Table S2. The e-value of the association between ASFDDS and PCS

Table S3. The association of physical component score (PCS) and mental component score (MCS) with animal source food diet diversity score (ASFDDS) based on logistic regression models (excluding participants with hypertension, diabetes, and pulmonary heart disease, n = 25296)

Table S4. The association of physical component score (PCS) and mental component score (MCS) with animal source food diet diversity score (ASFDDS) based on logistic regression models with all covariates without selection (adding drinking, smoking, ODDS, staple food, and sleeping problems in model 3)

Table S5. The association of physical component score (PCS) and mental component score (MCS) with animal source food diet diversity score (ASFDDS) based on logistic regression models with unfilled data (n = 35783)

Figure S1. The flow chart for study participant selection

Figure S2. A priori-defined directed acyclic graph

Figure S3. Subgroup analyses for physical component score (PCS), mental component score (MCS), and animal source food diet diversity score (ASFDDS)

Figure S4. The association between sequential exclusion of each food from the ASFDDDS and PCS/MCS

Table S1. The characteristics of the participants included and excluded

	**Inclusion n = 39997**	**Exclusion n = 8028**	χ^2/F	***P***
gender—men, n (%)	14383 (36.0)	4214 (52.5)	770.02	<0.001
Age (year), $\bar{x} \pm s$	50.43 ± 13.09	43.18 ± 13.89	2012.57	<0.001
Age group, n (%)				
≤40	9008 (22.5)	3728 (46.4)	2555.46	<0.001
41–50	8181 (20.5)	1929 (24.0)		
51–60	11895 (29.7)	1269 (15.8)		
≥61	10912 (27.3)	1102 (13.7)		
SES, n (%)				
Low	16628 (42.2)	516 (6.8)	5455.63	<0.001
Median	14191 (36.0)	2545 (33.6)		
High	8594 (21.8)	4523 (59.6)		
Married, n (%)	35233 (88.5)	6607(84.3)	108.71	<0.001
Urban, n (%)	12021 (30.1)	7126 (88.7)	9612.85	<0.001
Drinking, n (%)	3238 (8.1)	1554 (20.0)	2767.75	<0.001
Smoking, n (%)	5996 (15.1)	1669 ((21.9)	485.03	<0.001
BMI (kg/m^2), $\bar{x} \pm s$	23.82 ± 3.57	23.93 ± 3.34	6.46	0.011
BMI group, n (%)				
<18.5	1582 (4.7）	248 (3.8)	19.04	<0.001
18.5–23.9	14293 (42.8)	2637 (41.2)		
≧24.0	17495 (52.5)	3523 (55.0)		
History of chronic disease, n (%)	14507 (36.8)	2247 (32.3)	50.59	<0.001
Physical activity (MET:h/d), $\bar{x} \pm s$	21.94 ± 12.69	30.22 ± 11.43	873.56	<0.001
Frequency of staple food intake (times/week), $\bar{x} \pm s$	11.58 ± 4.51	11.87 ± 5.49	15.68	<0.001
Unhealthy eating habits (times/week), $\bar{x} \pm s$	3.80 ± 6.20	7.17 ± 8.60	754.97	<0.001
ODDS, $\bar{x} \pm s$	2.00 ± 0.93	2.20 ± 1.07	453.714	<0.001

Table S2. The e-value of the association between ASFDDS and PCS

	OR (95%CI)	E-value
**Total**		
ASFDDS as a continuous variable	1.09 (1.04, 1.14)	1.40
ASFDDS falls into three categories	1.26 (1.13, 1.40)	1.83
**In Men**		
ASFDDS as a continuous variable	1.08 (1.01, 1.15)	1.37
ASFDDS falls into three categories	1.16 (1.01, 1.34)	1.60
**In women**		
ASFDDS as a continuous variable	1.10 (1.04, 1.16)	1.43
ASFDDS falls into three categories	1.34 (1.16, 1.54)	2.01

E-value analysis

The e-value, which is mainly used to evaluate the influence of unmeasured confounding on the obtained results, is simple to calculate and has been widely used in the sensitivity analysis of observational studies. The main idea is assuming that there is an unmeasured confounding, then at least how strong is the association between this unmeasured confounding and expose-outcome to completely offset the current relationship between expose-outcome. Similar to the P-value, the e-value can be used to assess the robustness of the results to potential unmeasured confounders, thus providing strong evidence of causality.

$$\mathrm{E-value} = \mathrm{OR} + \sqrt{\mathrm{OR} \times (\mathrm{OR} - 1)}$$

Table S3. The association of physical component score (PCS) and mental component score (MCS) with animal source food diet diversity score (ASFDDS) based on logistic regression models [1] (excluding the participants with hypertension, diabetes, and pulmonary heart disease, n = 25296)

	**ASFDDS as a continuous variable**		**ASFDDS = 0**	**ASFDDS = 1**		**ASFDDS ≥ 2**	
	**OR (95%CI)**	***P***	**Ref.**	**OR (95%CI)**	***P***	**OR (95%CI)**	***P***
Total							
PCS	1.12 (1.08, 1.16)	<0.001	Ref.	1.11 (1.00, 1.24)	0.042	1.33 (1.18, 1.50)	<0.001
MCS	1.06 (1.01, 1.12)	0.028	Ref.	0.99 (0.88, 1.11)	0.822	1.19 (1.02, 1.38)	0.023
Men							
PCS	1.11 (1.04, 1.18)	0.001	Ref.	1.01 (0.85, 1.21)	0.873	1.26 (1.03, 1.54)	0.022
MCS	1.11 (1.03, 1.20)	0.008	Ref.	1.06 (0.88, 1.29)	0.544	1.38 (1.09, 1.74)	0.008
Women							
PCS	1.12 (1.07, 1.08)	<0.001	Ref.	1.17 (1.03, 1.34)	0.016	1.36 (1.17, 1.59)	<0.001
MCS	1.02 (0.95, 1.10)	0.561	Ref.	0.95 (0.82, 1.10)	0.949	1.08 (0.89, 1.31)	0.442

[1] PCS/MCS as the dependent variable were categorized by decile, using the highest decile compared with the other deciles in the logistic regression model. The analysis was adjusted for age, gender (in total analyses), SES, marital

status, residence, physical activity, history of chronic disease, unhealthy eating habits, and BMI.

Table S4. The association of physical component score (PCS) and mental component score (MCS) with animal source food diet diversity score (ASFDDS) based on logistic regression models with complete covariates[1]

	ASFDDS as a continuous variable		ASFDDS = 0 (n = 17445)	ASFDDS = 1 (n = 13022)		ASFDDS ≥2 (n = 9530)	
	**OR (95%CI)**	***P***	**Ref.**	**OR (95%CI)**	***P***	**OR (95%CI)**	***P***
Total							
PCS	1.09 (1.05, 1.13)	<0.001	Ref.	1.07 (0.97, 1.17)	0.178	1.21 (1.08, 1.35)	0.001
MCS	1.02 (0.97, 1.06)	0.492	Ref.	0.97 (0.89, 1.06)	0.466	1.07 (0.95, 1.20)	0.277
Men							
PCS	1.07 (1.01, 1.14)	0.018	Ref.	1.04 (0.91, 1.17)	0.125	1.12 (1.00, 1.28)	0.054
MCS	1.03 (1.02, 1.03)	0.080	Ref.	1.04 (0.90, 1.20)	0.634	1.18 (0.99, 1.41)	0.060
Women							
PCS	1.10 (1.05, 1.16)	<0.001	Ref.	1.14 (1.02, 1.28)	0.026	1.26 (1.09, 1.45)	0.002
MCS	0.98 (0.93, 1.04)	0.557	Ref.	0.93 (0.83, 1.04)	0.227	1.00 (0.86, 1.17)	0.983

[1] PCS/MCS as the dependent variable were categorized by decile, using the highest decile compared with the other deciles in the logistic regression model. The analysis was adjusted for age, gender (in total analyses), SES, marital status, residence, physical activity, BMI, history of chronic disease, unhealthy eating habits, drinking, smoking, other DDS, staple foods, and sleeping problems.

Table S5 The association of physical component score (PCS) and mental component score (MCS) with animal source food diet diversity score (ASFDDS) based on logistic regression models with unfilled data (35783)[1]

	ASFDDS as a continuous variable		ASFDDS = 0	ASFDDS = 1		ASFDDS ≥2	
	**OR (95%CI)**	***P***	**Ref.**	**OR (95%CI)**	***P***	**OR (95%CI)**	***P***
Total							
PCS	1.09 (1.08, 1.11)	<0.001	Ref.	0.96 (0.89, 1.04)	0.350	1.23 (1.12, 1.34)	<0.001
MCS	1.08 (1.07, 1.10)	<0.001	Ref.	0.95 (0.88, 1.02)	0.164	1.21 (1.10, 1.33)	<0.001
Men							
PCS	1.08 (1.06, 1.10)	<0.001	Ref.	0.86 (0.76, 0.98)	0.023	1.16 (1.01, 1.34)	0.038
MCS	1.12 (1.09, 1.14)	<0.001	Ref.	0.98 (0.87, 1.11)	0.746	1.31 (1.13, 1.52)	<0.001
Women							
PCS	1.10 (1.09, 1.12)	<0.001	Ref.	1.03 (0.93, 1.13)	0.586	1.25 (1.12, 1.41)	<0.001
MCS	1.06 (1.04, 1.08)	<0.001	Ref.	0.94 (0.84, 1.05)	0.258	1.04 (0.89, 1.21)	0.641

[1]PCS/MCS as the dependent variable were categorized by decile, using the highest decile compared with the other deciles in the logistic regression model. The analysis was adjusted for age, gender (in total analyses), SES, marital

status, residence, physical activity, history of chronic disease, unhealthy eating habits, and BMI.

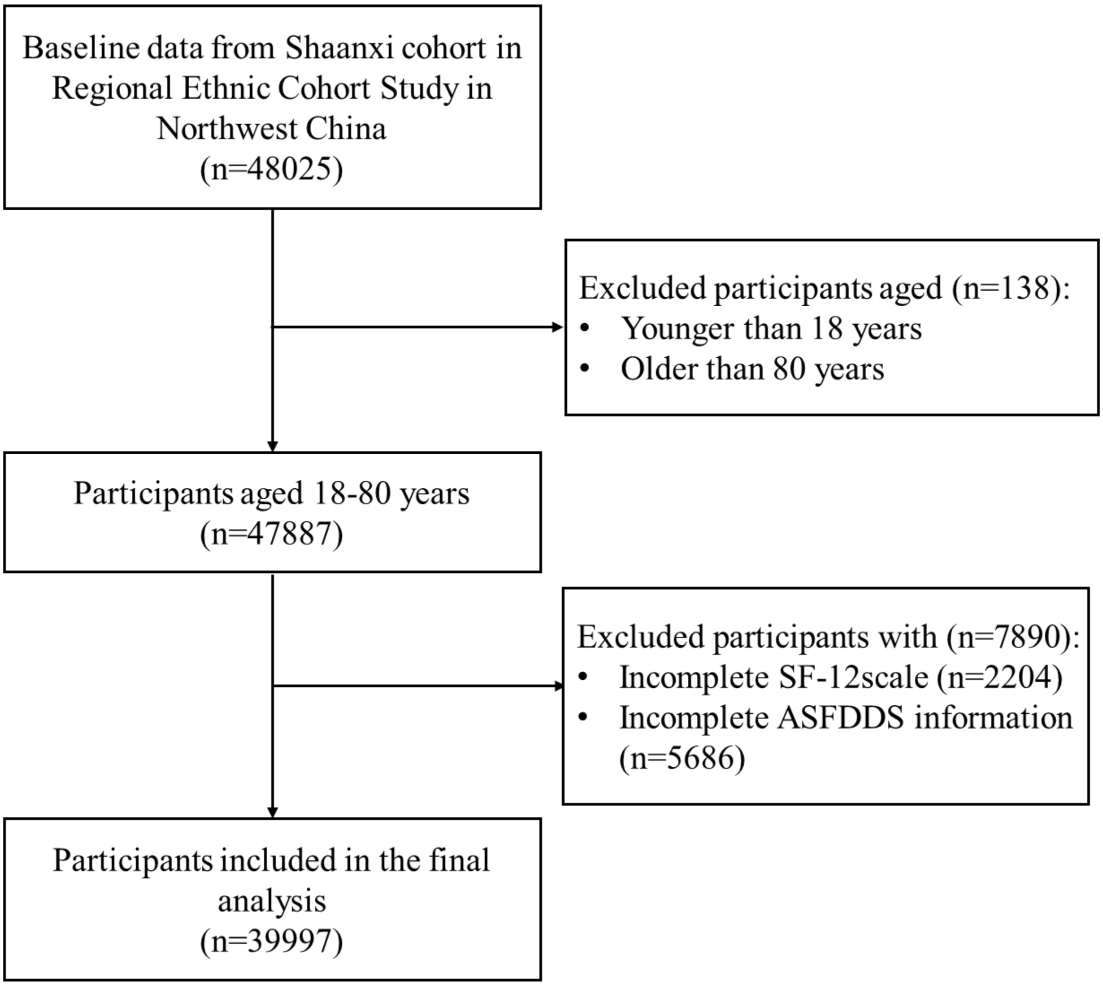

Figure S1. The flow chart for study participant selection

Directed Acyclic Graph

The Directed Acyclic Graph (DAG) was used to identify the causal relationship among the 10 confounding variables based on existing professional knowledge. DAG uses graph recognition to control the minimum sufficient adjustment set of confounding factors, avoiding excessive adjustment, which is conducive to more accurate exploration of the relationship between variables. Finally, “smoking, drinking, ODDS, staple food and sleeping problem” were excluded when covariables were adjusted in this study because these variables were in the causal pathway between other variables and HRQoL.

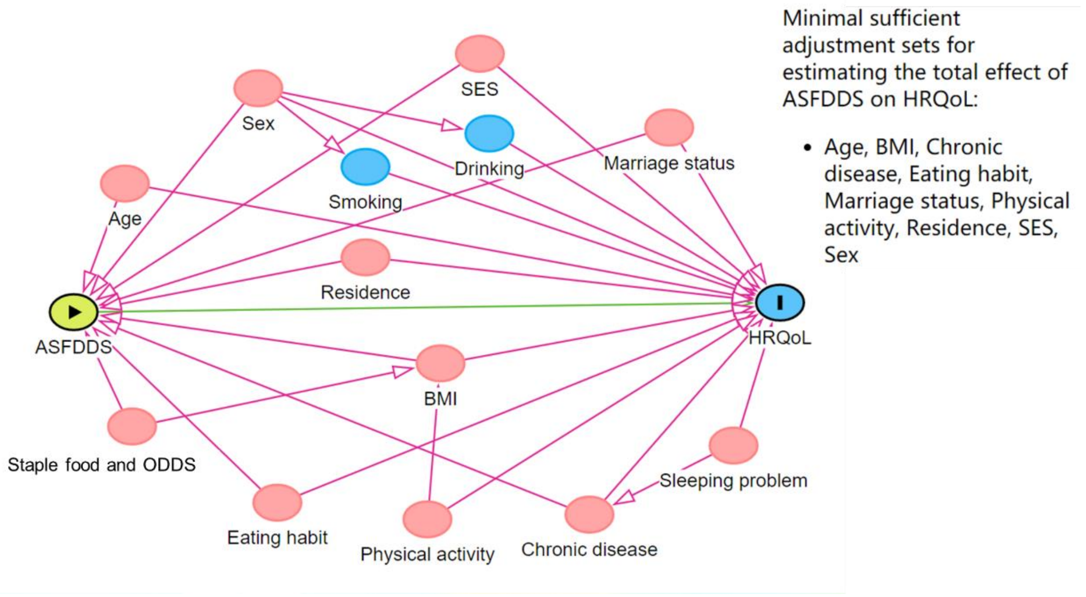

Figure S2. A priori-defined directed acyclic graph

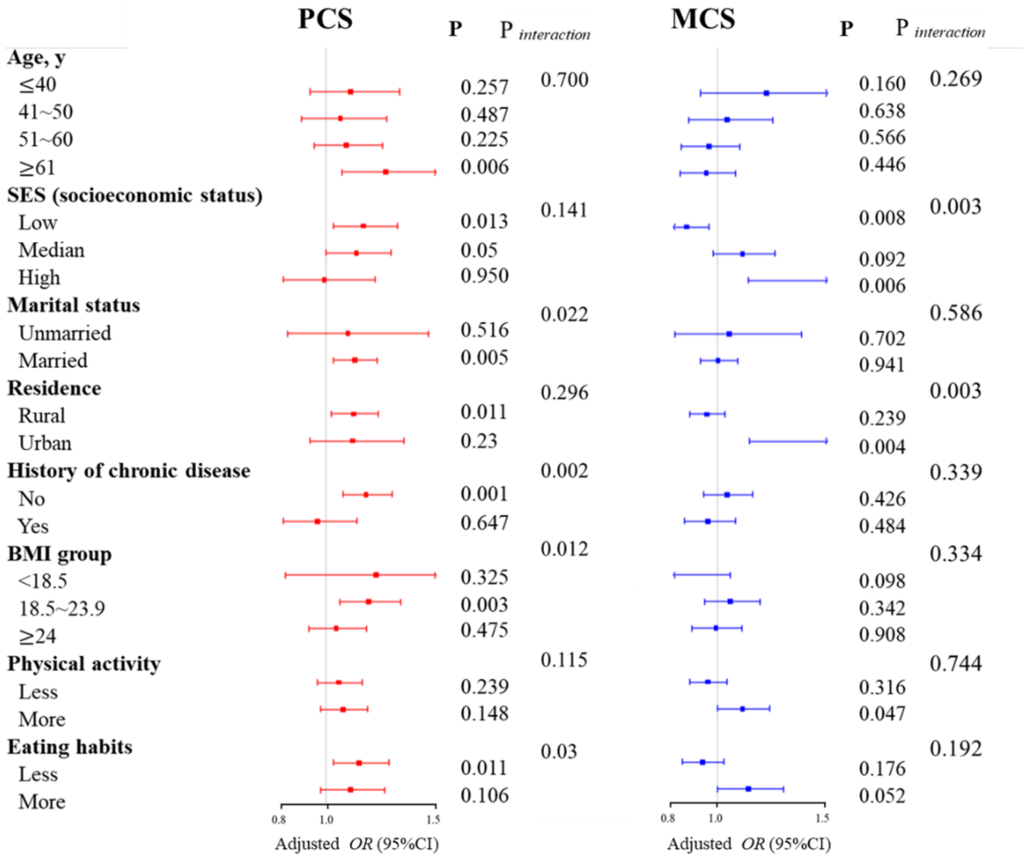

Figure S3. Subgroup analyses for physical component score (PCS), mental component score (MCS), and animal source food diet diversity score (ASFDDS)

Note: All results were adjusted for variables other than the subgroup variables. Interaction effects were tested for each subgroup.

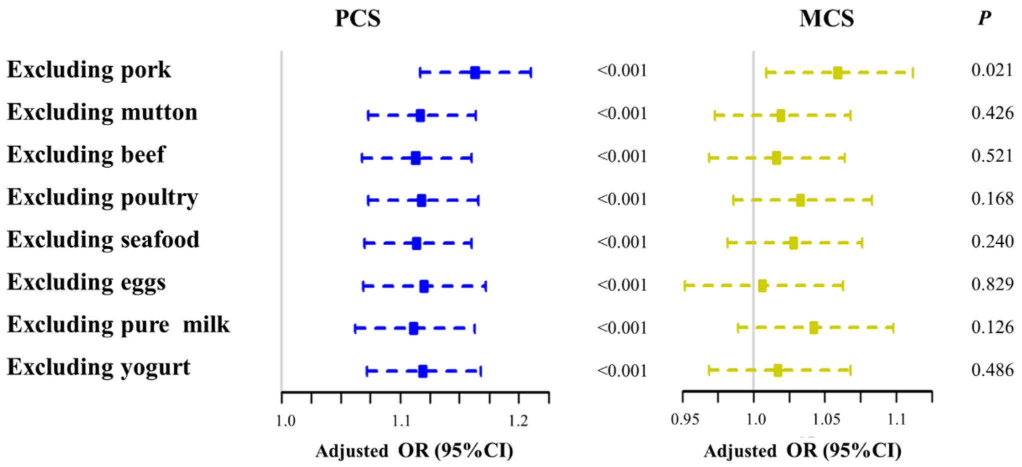

Figure S4 The association between the sequential exclusion of each food from the ASFDDDS and PCS/MCS

Note: all results are adjusted for age, gender, SES, marital status, residence, physical activity, history of chronic disease, unhealthy eating habits, and BMI.
